# The association between psychological burden related to COVID-19 and addictive social media use: Testing the mediational role of anxious affect

**DOI:** 10.1371/journal.pone.0271332

**Published:** 2022-07-12

**Authors:** Zahir Vally, Mai Helmy

**Affiliations:** 1 Department of Clinical Psychology, United Arab Emirates University, Al Ain, UAE; 2 Wolfson College, University of Oxford, Oxford, United Kingdom; 3 Department of Psychology, Sultan Qaboos University, Muscat, Sultanate of Oman; 4 Department of Psychology, Menoufia University, Shebin El-Kom, Egypt; Charles Darwin University, AUSTRALIA

## Abstract

**Introduction:**

The COVID-19 pandemic has had a substantial impact on the normalcy of life. Similarly, social media use (SMU) has increased exponentially. This study examined the association between individuals’ perception of the psychological burden related to the pandemic and addictive SMU.

**Method:**

A cross-sectional study was conducted between February and May 2021 in two national contexts, Egypt, and the United Arab Emirates. Data were gathered from a sample of 1322 participants drawn from a university population who completed measures of psychological burden related to COVID-19, anxious affect, and addictive SMU. Preliminary analyses of the potential association between the study variables were conducted using bivariate correlations followed by a pre-specified mediation model.

**Results:**

At a correlational level, all three study variables were positively associated with each other (r values ranged from .18 to .50 and all p values were < .05). A further mediation analysis confirmed that the total effect of psychological burden on addictive SMU was significant (β = .654, SE = .033, 95% CI .589-.720), and this relationship remained significant with inclusion of the mediator. Significant mediation was evident across the total sample as well as within each country-specific subsample.

**Conclusion:**

These results provide insight into the factors that contribute to the development of addictive SMU in relation to the COVID-19 pandemic. Findings are discussed in relation to the emotion regulation function that SMU might play for individuals in the midst of emotional distress.

## Introduction

The outbreak of coronavirus in 2019 (COVID-19) and its consequent spread across the globe has had a substantial and varied impact on the lives of every individual on the planet [1]. Many people have perceived the pandemic as a heavy burden to endure and the magnitude of this sense of psychological burden has continued to accrue as individuals now negotiate the aftermath of this experience [2]. As countries emerge from periods of lockdown; it should not be assumed that the experienced difficulties of the lockdown period will dissipate. There is some evidence that suggests that the prevalence of depressive disorders, for example, may in fact be higher now than compared to the peak of the periods of lockdown during 2020 [3].

Individuals have reported experiencing a subjective loss of control, an experience that appears to be accompanied by immense anxiety [4, 5]. Individuals’ experience of anxiety may be related to fear of becoming infected and concern about the risk of this occurring. They may be anxious about the anticipated future consequences of the pandemic, these concerns may be related to job security and financial stability, political instability, or the impact on their interpersonal relationships [6, 7].

A consideration of individuals’ social media use (SMU) is relevant to the current study given that research suggests that elevated levels of anxious affect and the subjective lack of control that appears to accompany anxiety is related to engagement in excessive SMU [8–12]. Additionally, recent research suggests that, since the COVID-19 pandemic, a noticeable increase in SMU is evident [13]. For some individuals, use of social media during times of immense distress may serve an adaptive function. It serves as a means of escape from the real-world and thus avoidance of the distressing situation being faced [14]. It may also enable the individual to regain a subjective sense of control in the virtual realm in the midst of otherwise unpredictable life experiences [12, 15]. In relation to the COVID-19 pandemic, some individuals may engage in intensive SMU to mitigate the reduced sense of control in the real world and to escape the anxiety caused by the unpredictability of the pandemic [9, 10, 12]. Initially, SMU can reduce feelings of loneliness and isolation and enable individuals to elicit social support from others in the virtual world [16]. This is especially relevant as individuals struggle with being cut off from others when engaging in spatial distancing and working from home. However, there is the possibility that this may precipitate a dysfunctional emotional need for constant and excessive engagement in SMU as individuals come to rely upon it as an emotion regulation mechanism [10]. This may contribute to the development of addictive tendencies and negatively impact overall mental health [17–19].

Neither the Diagnostic and Statistical Manual of Mental Disorders [20] or the International Classification of Diseases manual [21] presently recognize addictive SMU as a diagnosis. However, a burgeoning body of research has rapidly accumulated that attest to the phenomenon’s prevalence and the deleterious physical and mental health consequences that appear to accompany it [14, 22–24]. Research suggests positive associations between addictive SMU and the experience of stress, depression, and anxiety [8, 23]. Moreover, when assessed longitudinally in a cohort of participants with mood disorders, baseline levels of addictive SMU predicted individuals’ level of depressive symptoms and insomnia at the second assessment point six weeks later [25]. In a college-aged population, baseline levels of addictive SMU were predictive of engagement in suicidal ideation and behaviour one year later [19]. Therefore, it appears that when individuals engage in excessive SMU as a means of compensating for a perceived lack of control, this may serve an adaptive function in the short-term, but prolonged and increasing reliance on SMU as a coping strategy clearly contributes to the development of unfavourable mental health consequences.

There is also evidence that demonstrates a significant relationship between SMU and the emergence of anxious affect [2, 9, 18]. The argument typically provided in explanation of this association is that the content posted to social media platforms provide exposure to unfiltered information, potentially misinformation, that may be particularly overwhelming during times of societal distress such as an outbreak of infectious disease [2]. Recent research conducted specifically in relation to participants’ experience of COVID-19 information demonstrates a similar relationship [13]. Despite the frequent claims by social media companies that they regularly engage in deliberate removal of misinformation posted to their platforms, this action may not always be uniform (across countries and regions) and may not necessarily be swift enough to prevent some users from having viewed it. Thus, the exposure to unfiltered misinformation and users’ virtual interaction with it may contribute to what Brailovskaia and Margraf [2] refer to as an ‘emotional contagion’ and thus to a precipitous exacerbation of anxious affect.

**Fig 1:**
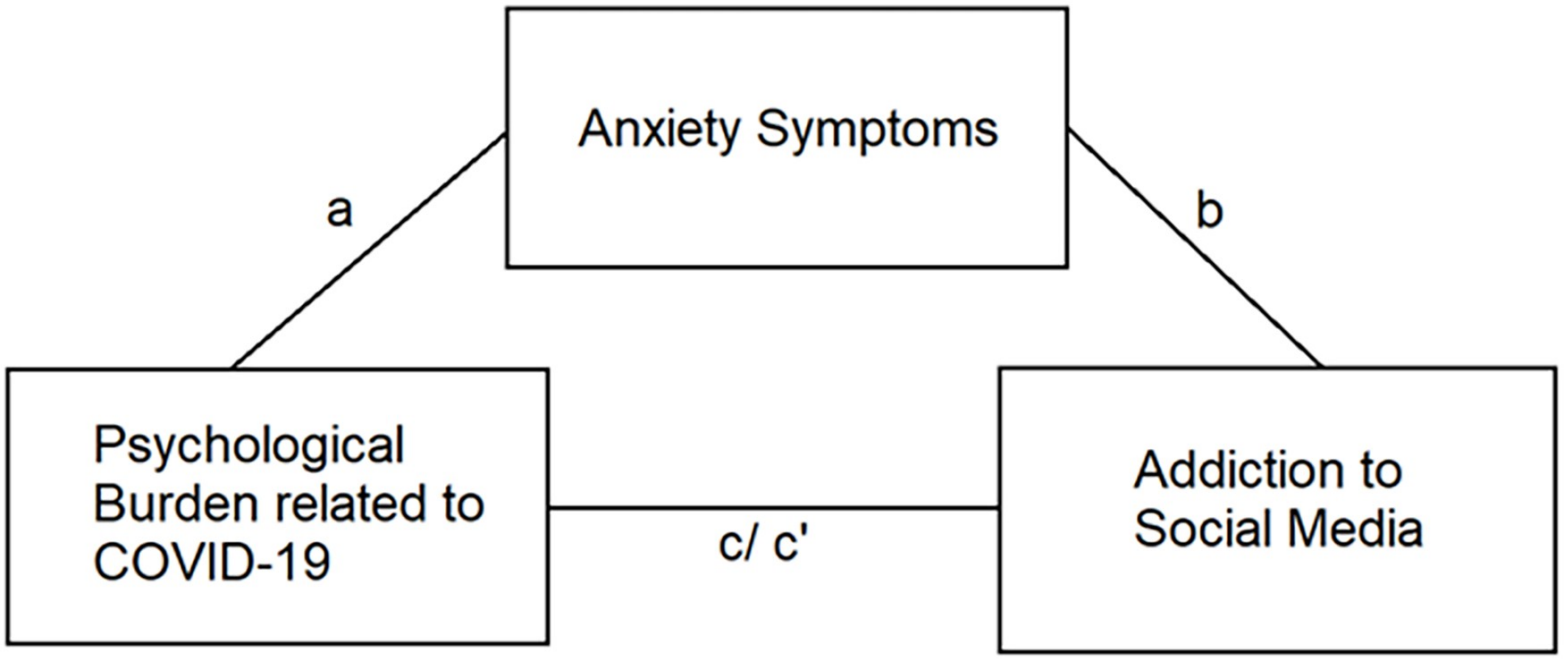
Proposed mediation model consisting of psychological burden related to COVID-19 as the predictor, anxiety as the proposed mediator, and social media addiction as the outcome. a = path of predictor to mediator; b = path of mediator to outcome; c = path of predictor to outcome without mediator; c’ = path of predictor to outcome with inclusion of the mediator.

Whilst many countries have begun to return to a modified form of normalcy, the psychological burden that people experience is likely to persist as they navigate the negative ramifications of the pandemic. Research has demonstrated that psychological burden caused by stressful life experiences diminishes individuals’ perception of their sense of control and this can contribute to the onset of mental health difficulties [26]. Brailovskaia and Margraf [2] define psychological burden related to COVID-19 as the experience of being overwhelmed by the pandemic, generally, but also more specifically by the imposition of the variety of restrictions to the normalcy of daily life. The construct also includes the experience of a loss of control of over one’s present-day life and one’s future. The primary aim of the present study was to examine the association between psychological burden related to COVID-19 and addictive SMU. We propose the following hypotheses. First, psychological burden related to COVID-19 will be positively associated with addictive SMU (Hypothesis 1a). Anxiety is expected to be positively associated with both psychological burden (Hypothesis 1b) and addictive SMU (Hypothesis 1c). Then, anxiety is expected to mediate the association between psychological burden and addictive SMU (Hypothesis 2). Fig 1 illustrates the hypothesized relationships in the proposed mediation model.

## Materials and methods

### Study design and setting

A cross-sectional study was implemented in which data were collected from university students during the Spring semester of 2021 at the campuses of the United Arab Emirates University and Menoufia University in Egypt. Sampling from these two locations was driven principally by convenience. However, these two countries provide data that facilitate comparisons to be made in respect of the research questions. At the time of data collection, strict lockdown measures were in place in the United Arab Emirates (UAE) (e.g., movement between cities was restricted, work-from-home orders and home schooling was still in place, and fines instituted for violations of the rules), whilst, in Egypt, a less restrictive approach had been adopted (e.g., mask-wearing was not enforced, and schools and universities remained open). Comparative data from these two countries allows us to examine the impact of the divergent management of the pandemic in these two settings on the study’s research questions.

### Ethics

The Social Sciences Research Ethics Committee at the United Arab Emirates University (reference number: ERS_2020_6102) provided ethical permission for the conduct of this study. All participants provided written informed consent prior to study participation. Participants were provided with a participant information sheet outlining the responsibilities of the research team and their rights as participants (e.g., confidentiality, right to withdraw without penalty).

### Study sample, sampling techniques, and recruitment

The participants included in this study were recruited from two different countries in the Middle East, Egypt, and the UAE. Inclusion criteria for participation included adults, aged 18 years old and above, who were current users of social media (this entailed having an account on any social media platform). Participants were recruited using announcements that were made in the classes taught by the two researchers. A combined total of approximately 2000 participants across these two locations formed the potential sampling frame. These students were also requested to promote the study elsewhere on their social media feeds and amongst their friends and families. We also placed virtual advertisements on the university’s social media accounts as these would enable access to other students on the campuses. Students completed an electronic survey using a link that was made available to all participants who elected to participate. The access link was also included in the advertisements for the study. The study was implemented from February to May 2021.

### Assessment measures

#### Psychological burden related to COVID-19

We employed the measure developed by Brailovskaia and colleagues [2, 10] to assess the extent to which individuals experienced psychological burden related to the COVID-19 pandemic. The measure consists of 6 items (Example items include “I feel socially isolated” and “I am burdened by the current social situation”) to which respondents provide a response using a 4-point Likert scale that ranges from 0 (I do not agree) to 3 (I totally agree). Greater levels of experienced psychological burden are indicated by higher cumulative scores. The measure has previously produced average to good internal consistency across a number of subsamples in a multi-country study (α = .65 to .77) [2]. In the present sample, Cronbach’s alpha was .74, which indicates satisfactory internal consistency.

#### Anxiety

The anxiety subscale of the Depression Anxiety Stress Scales 21 (DASS-21) [27] was used to measure general anxious affect over the preceding week using seven items in which participants provide a rating using a 4-point Likert-type scale that ranges from 0 (*did not apply to me at all*) to 3 (*applies to me very much or most of the time*). A higher total score following the combination of the ratings across the seven items is indicative of elevated levels of anxiety. Example items include “I experienced trembling in the hands” and “I felt scared without any good reason”. The DASS-21 is one of the most widely used measures of depressive and anxious affect. A substantial range of evidence has been generated attesting to its reliability and validity. It has been used, reliably, across a wide range of participants including amongst college-aged young adults [28]. The Arabic version of the DASS-21, in particular, is theoretically coherent and has been shown to possess high construct, convergent, and discriminant validity [29]. For the Arabic version, Cronbach’s alpha values typically range from .88 to .93 [29–31]. Internal consistency in the present study was similarly high (α = .81).

#### Addictive social media use

The abbreviated, six-item version of the Bergen Social Media Addiction Scale (BSMAS) [17] was used to assess addictive SMU. This brief version was developed based upon the Bergen Facebook Addiction Scale [32] and was designed to target the six core features of addiction (i.e., salience, tolerance, mood modification, relapse, withdrawal, conflict). Respondents provide ratings to the six items using a 5-point Likert-type scale that ranges from 1 (*very rarely*) to 5 (*very often*) with higher total scores indicating higher levels of addictive SMU. The BSMAS has been shown to possess a unidimensional factor structure and adequate reliability and validity [33, 34]. Internal consistency in the present sample was good (α = .80).

### Data analysis

Descriptive analyses were conducted, and the results of these analyses are reported using means and standard deviations for continuous variables or counts and percentages for categorical variables. As a means of providing a preliminary indication of the potential association between psychological burden related to COVID-19, anxiety symptoms, and addictive SMU, bivariate correlations were conducted, and the results of these analyses are reported using Pearson’s correlation coefficients. Then, a pre-specified mediation model was subjected to analysis. This included psychological burden related to COVID-19 as the predictor variable, anxiety as the potential mediator, and addictive SMU as the outcome variable. Additionally, because age and gender have been shown to be related to addictive SMU [23], these were included as covariates in the model. The proposed mediation model is depicted in Fig 1. In the illustrated model, path a indicates the relationship between psychological burden and anxiety, path b represents the association between anxiety and addictive SMU. The indirect effect (ab) is illustrative of the combined effect of both paths a and b. The total effect, of the association between psychological burden and addictive SMU, is represented as path c, whilst the relationship between these two variables (the predictor and outcome) following inclusion of the proposed mediator (anxiety), is denoted as path c’ (the direct effect). This mediation analysis employed a bootstrapping procedure (10,000 samples) that results in bootstrapped confidence intervals (95%CI). All analyses were conducted in both the total sample as well as for each country. Formative analyses were conducted using SPSS 26 and the mediation analyses were conducted using the PROCESS macro version 3.5 (www.processmacro.org/index.html) [35]. A p value of .05 was regarded as statistical significance in all analyses.

## Results

### Descriptive and correlational analyses

A sample of 1322 participants (m_age_ = 19.50 years, SD = 1.54) was recruited. The sample was primarily comprised of students recruited from the campuses of the researchers’ universities (96.4%). This was expected given the targeted recruitment strategies employed. A small proportion of the sample were currently in fulltime employment whilst studying on a part-time basis (2.6%) and the remaining 1% were unemployed. The majority of the sample were single (90.6%) and female (75.4%). Egyptian participants totalled 1036 whilst 286 participants were from the UAE. Despite a substantial preponderance of Egyptian participants in the sample compared to UAE participants (78.4% versus 21.6% for the Egyptian and UAE participants, respectively), the comparative distribution of the demographic variables did not significantly differ between the two countries. Table 1 depicts these comparative values for the demographic variables.

**Table 1:** Descriptive statistics for demographic variables (total sample and for each country).

	Total n = 1322	Egypt n = 1036	UAE n = 286
Age M (SD)	19.50 (1.54)	19.45 (1.43)	19.69 (1.88)
Gender (Female %)	75.4	74.1	80.1
Marital status %			
Single	90.6	91.0	89.2
In a relationship	7.0	6.6	8.4
Married	2.4	2.4	2.4
Occupation %			
Fulltime student	96.4	96.7	95.1
Part-time student and employed	2.6	2.2	4.2
Recently graduated and unemployed	1.0	1.1	0.7

*Note*. M = mean; SD = standard deviation; UAE = United Arab Emirates.

Table 2 illustrates the descriptive results for each of the study variables in both the overall sample as well as for each of the country-stratified subsamples. When the mean scores for each study variable were compared between the two countries, no significant differences were evident (all p > .05). Correlational analyses indicated statistically significant associations between all the study variables and across all possible points of comparison (all p < .01). The magnitude of the Pearson’s r values ranged from .18 to .50 (see Table 2).

**Table 2:** Descriptive results and bivariate correlations between the study variables.

	M (SD)	Min-Max	2	3
*Overall sample n = 1322*				
1. Psychological burden	7.85 (3.69)	0–20	.29**	.48**
2. Anxiety	2.53 (3.09)	0–24	-	.22**
3. SMA	11.81 (5.07)	0–18	-	-
*Egypt n = 1036*				
1. Psychological burden	7.83 (3.71)	0–20	.31**	.47**
2. Anxiety	2.54 (3.13)	0–24	-	.24**
3. SMA	11.86 (5.02)	0–18	-	-
*UAE n = 286*				
1. Psychological burden	7.94 (3.65)	0–20	.18**	.50**
2. Anxiety	2.41 (2.95)	0–24	-	.18**
3. SMA	11.61 (5.27)	0–18	-	-

*Note*. M = mean, SD = standard deviation, UAE = United Arab Emirates, SMU = social media addiction,

**p < .01 (2-tailed).

### Mediation analyses

Results of the mediation analyses were as follows. First, when the total sample was considered, the overall model was significant (F(2, 1319) = 202.768, R^2^ = .235, p < .001). Moreover, the total effect of psychological burden on addictive SMU was significant (β = .654, SE = .033, 95% CI .589-.720), and this relationship remained significant with inclusion of the mediator in the analysis. However, the magnitude of the coefficients decreased (.654 in path c and .617 in path c’) suggesting that the mediator, anxiety, partly mediated the association between burden and addictive SMU. A similar pattern of results was evident in the Egyptian subsample. The total and indirect effects (paths c and c’) were both significant and a reduction in the coefficients was evident when the mediator was included (.636 versus .594) suggesting the presence of a partial mediational effect. When the UAE sample was examined, the total effect was significant, reflective of an association between psychological burden and addictive SMU, and this relationship remained statistically significant when anxiety was included as a potential mediator (the direct effect). The indirect effect (path ab) was not significant as the confidence intervals crossed the zero-threshold suggesting that the included mediator contributed substantially to the association of psychological burden and addictive SMU. Table 3 summarizes the total, direct and indirect effects of these mediation analyses.

**Table 3:** Estimated coefficients of the tested mediation models, overall sample and for each country (outcome: Social media addiction).

	Total Effect			Direct Effect			Indirect Effect		
	c	SE	95%CI	c’	SE	95%CI	ab	SE	95%CI
Overall sample									
SMA	.654	.033	.59-.72	617	.035	.55-.69	.037	.011	.02-.06
Egypt									
SMA	.636	.037	.56-.70	.594	.039	.52-.67	.042	.013	.02-.07
UAE									
SMA	.727	.074	.58-.87	.703	.075	.56-.85	.024	.017	-.003-.06

*Note*. UAE = United Arab Emirates, SMU = social media addiction, SE = standard error, 95%CI = confidence interval computed bootstrapping at 10,000 samples, c = relationship between psychological burden and addictive social media use, c’ = relationship between psychological burden and addictive social media use with the inclusion of anxiety in the model, ab = combined effect of paths a and b.

## Discussion

This study investigated the mental health difficulties experienced by individuals during the COVID-19 pandemic. We assessed psychological burden experienced during the pandemic and the extent to which this was related to the development of addictive SMU. Additionally, our data contributes to an understanding of the role played by anxious affect in this relationship. In confirmation of hypothesis 1a, psychological burden related to COVID-19 was positively associated with addictive SMU. Our data also confirmed hypotheses 1b and 1c as anxiety emerged as significantly related to both psychological burden as well as addictive SMU. When the potential mediational role of anxiety was examined, anxiety significantly mediated the association between psychological burden and addictive SMU (confirming hypothesis 2). Thus, it appears that for individuals who subjectively experience the COVID-19 pandemic as especially burdensome, elevated levels of anxious affect is likely, and this in turn, increases the degree to which excessive and addictive use of social media platforms will ensue.

Skaff’s [26] work provides a relevant context within which to conceptualize these findings. It was proposed that situations and environments that are experienced as being burdensome reduce an individuals’ sense of control over the experience itself as well as their lives in general. Research also suggests that individuals who believe that they possess minimal control over their lives and thus cannot exert volitional behaviour to bring about a sense of certainty may suffer from a range of unfavourable mental health consequences. Depression, anxiety, and burnout appears to be common consequences [36]. They are also more likely to develop maladaptive coping strategies in an attempt to exert some degree of control and these typically manifest as dysfunctional symptomatic behaviours such as substance abuse, or restrictive or binge eating [37, 38].

Our results support the validity of Skaff’s [26] contentions and suggests that the theory’s proposition may also apply to the COVID-19 experience. Evidence suggests that individuals do indeed experience the pandemic as burdensome [2, 25, 39]. The typical routines that characterize daily life were inverted and were followed by being required to adhere to strict rules that circumvented the normalcy of daily life. Many individuals felt that they were no longer able to exert control over their lives [4, 6]. Research suggests that when individuals find themselves in uncertain situations, they are likely to divert their attention towards alternate avenues in which they might potentially regain a semblance of control. They may thus engage in online interactions on social media platforms where there is the possibility to control one’s actions (e.g., what content to post or not post, how one chooses to present oneself, or with whom to interact) [40]. Additionally, the diversion to working and schooling from home resulted in people having more free time than they typically would have if engaged in fulltime work or study and this precipitated a substantial surge in the frequency and intensity of SMU [13].

Excessive SMU may also serve an emotion regulation function. For those who experience emotional distress, excessive SMU may facilitate the pursuit of positive emotional states and, conversely, the remediation of negative emotional states [41]. Research clearly demonstrates the potential for SMU to combat loneliness [16], promote a sense of belonging, and facilitate social support [42], elements that invariably may be absent from the person’s real life. But the positively reinforcing experience derived from this form of SMU may result in an intense emotional bond to the device or the platform or, more specifically, to the function it serves [43, 44]. They may come to inordinately rely on social media for the positive experiences that appear to accompany its use and thus increased and excessive use may emerge. In a similar fashion to individuals who struggle with other forms of addictive behaviours such as substance abuse or overeating, individuals may experience a loss of control over their SMU [17]. This may result in a self-reinforcing cycle in which the individual attempts to regain the perception of control by excessively using social media and thus reinforcing the perception of self-control having been lost [14].

Our results also suggest that anxiety plays an important role as to whether and to what extent psychological burden is likely to contribute to addictive SMU—although, importantly, we cannot conclude whether this association is causal. More specifically, as anxiety levels appreciate, the association between perceived burden and addictive SMU is similarly amplified. The extent to which social media platforms are sources of positive reinforcement and of perceived social support may provide relief and emotional comfort for those struggling with anxiety related to the burden of their real-world experiences. This may promote an over-reliance on the platform as an emotion regulation mechanism [12].

### Strengths and limitations of the study

The findings of this study are timely given that the pandemic appears likely to continue, at least in an attenuated form, as individuals struggle with the consequences of the pandemic and the changes that it has exacted on their lives [39, 45]. Our analyses are based on a large sample increasing its reliability and the accuracy of our results. Most notably, the study’s findings hold clinical utility and could inform the development of possible strategies for targeting excessive SMU within the context of the pandemic. Specifically, where anxious affect presents and is related to a perceived loss of control, our results suggest that this construct would be a sound target for psychotherapeutic intervention. There are also a number of limitations to consider when interpreting our findings. First, our findings result from a cross-sectional design and thus allude to an association but do not allow for conclusions about potential causality between elevated perceived burden and mental health difficulties. Second, our sample was limited to college-aged young adults. This means that the findings relate squarely to this segment of the population and in these locations. Future studies might consider the inclusion of a more varied sample both in relation to age as well as gender. Third, timing is an important consideration when interpreting these findings. At the time of data collection (February to May 2021), the nature of the COVID-19 situation in these two countries were somewhat different, one experiencing immense restrictions and the other adopting a more liberal approach. Despite these differences in context, between country comparisons across all variables were not significantly different.

### Conclusion

In conclusion, our results contribute to the literature by providing evidence of the prevalence of psychological burden related to the COVID-19 pandemic. This is also one of the only studies to be conducted in this region of the world. Our results suggest that elevated levels of experienced burden about the pandemic may results in an increased tendency to develop addictive and excessive reliance on social media platforms. Moreover, the magnitude of this demonstrated relationship appears to be amplified by elevated levels of anxiety. Thus, individuals who experience the pandemic as burdensome and develop an anxious affect may be more likely to use social media platforms for the purpose of mood modification.

## Supporting information

S1 File Complete dataset for the present study (SPSS file). Study dataset.
(SAV)
